# Benign Enlargement of Subarachnoid Space in Infancy: "A Review with Emphasis on Diagnostic Work-Up"

**Published:** 2018

**Authors:** Nahid KHOSROSHAHI, Ali NIKKHAH

**Affiliations:** 1 Department of Pediatric Neurology, Bahrami Children's Hospital, Tehran University of Medical Sciences, Tehran, Iran; 2 Pediatric Neurology Research Center of Mofid Hospital, Shahid Beheshti University of Medical Sciences, Tehran, Iran

**Keywords:** Benign enlargement of subarachnoid space, External hydrocephalus, Macrocephaly, Infant

## Abstract

Macrocephaly is one of the most frequent reasons for referral to a pediatric neurologist. Benign enlargement of subarachnoid space (BESS) in infancy is the most common cause of macrocephaly and characterized clinically with large head circumference, normal or mildly motor and language delay and increased cerebrospinal fluid (CSF) in the subarachnoid space with normal ventricles or mild ventriculomegaly. In this review, we describe the etiology, epidemiology, clinical presentation, pathogenesis, neuroimaging, differential diagnosis, treatment and outcome of this entity from current literature with emphasis on diagnostic work-up.

## Introduction

Large head (Macrocephaly) means head circumference more than two standard deviations above the mean for age, sex, and body size, established using measurements and a standard growth chart (1). About 2% of normal population has macrocephaly (1, 2). The causes of a large head include hydrocephalus (an excess volume of CSF intracranially), megalencephaly (enlargement of the brain), thickening of the skull and hemorrhage or non-bloody fluid into the subdural or epidural spaces (1, 2). Macrocrania in infancy can be due to both benign and pathologic causes. Pathologic cases are rare, alternatively, benign macrocrania of infancy is more common (3). External hydrocephalus is the most common cause of macrocephaly in infants (2-4). It is a condition in infants and children with enlarged subarachnoid space accompanied by increasing head circumference with normal or mildly dilated ventricles (4, 5). 

Benign enlargement of subarachnoid space (BESS) encompasses a variety of names in literatures, such as benign external hydrocephalus (BEH), extraventricular hydrocephalus, benign subdural effusion, benign extracellular fluid collection, extraventricular obstructive hydrocephalus, subdural hygroma, pseudo-hydrocephalus, benign extra-axial collections, subarachnomegaly, and subdural effusions of infancy which demonstrating the confusion surrounding the entity (5-7).

**Figure 1:**
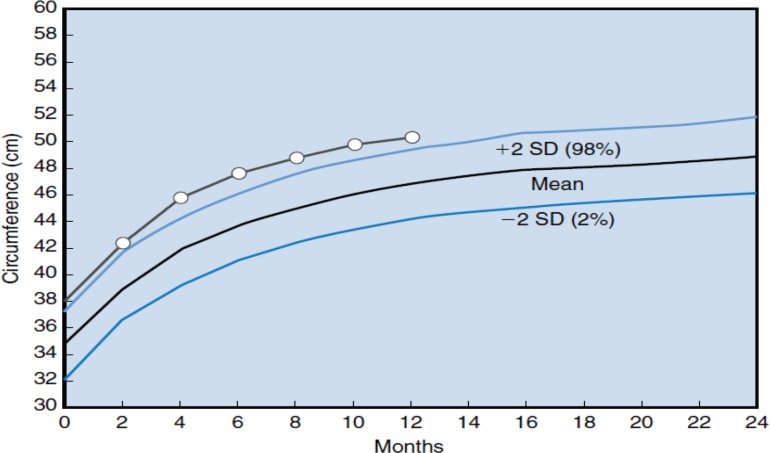
BESS. Head circumference is large at birth and grows above 98^th^ percentile (Fenichel Clinical Pediatric Neurology, 2013)

BESS is the most common cause of macrocephaly in infancy (4, 8, 9). It is more common in males (4, 10, 11). A genetic cause is likely in some cases, with the infants' father often having a large head (3, 12). Enlarging extra-axial fluid space is leading to an expansion of head circumference around 3 to 12 months of age, with head circumference measurement crossing percentile lines and often reaching above the 90^th^-98^th^ percentile (Figure 1) (2, 3, 13). Mean age at presentation was 7.3 months (14). Head circumference at birth is normal (2, 13, 15) or slightly higher than normal (14, 15).

## Etiology

Some causes for external hydrocephalus are mentioned but there is no definite cause for external hydrocephalus, therefor it is classified as idiopathic condition (2, 4). Hydrocephalus due to IVH, prematurity, meningitis, metabolic disorder, neurosurgery and trauma is not considered here (4, 5). About 40% of children with external hydrocephalus had at least one male person in their family close relative with macrocephaly (5, 8, 10, 11). This coherence was 80%-90% (2, 3). Autosomal dominant (3, 4, 15) and multifactorial model of inheritance have been assumed (4, 8, 16). 

## Epidemiology

An incidence of 0.4 per 1000 live births was reported only in one study (8). It is approximately 50% of hydrocephalic condition in retrospective and population based study in Norway (14). A review of incidental findings in a tertiary pediatric neurology center showed that 0.6% of the children had external hydrocephalus (15). 

## Historical notes

Initially intracranial fluid collections in infants were described in 1850s (17). The term benign external hydrocephalus (BEH) was first introduced in 1917 (7, 17, 18). Recently, the most usual name is BESS. 

## Clinical manifestations

The main feature of BESS is macrocephaly in a normal infant (2, 13-15). An otherwise normal infant is referred to medical attention because enlarging head size. Most studies report no signs and symptoms of increased intracranial pressure such as irritability, lethargy, vomiting, tense and bulging anterior fontanel (2, 3, 5). Rare studies reported a tense anterior fontanel (19, 20), dilated scalp veins (21), and frontal bossing (22). Sunset sign is not reported in any article (4). Neurologic findings are normal, but mild motor delay is often seen and final developmental status is often normal (2-4, 15, 16, 23).

## Pathophysiology

The most accepted theory about pathophysiology of external hydrocephalus is delayed maturation of the arachnoid villi not able to absorb the CSF produced continuously (3, 24). Expansion of subarachnoid space due to excessive amount of circulatory CSF is not leading to intracranial hypertension (24, 25). Maturation of arachnoid villi occurs in 18 months of age and the process was ended. There is the discrepancy between the skull and brain parenchymal growing which leading to a transient subarachnoid space enlargement (8, 26). On the other hand, external hydrocephalus may be associated with some conditions such as; hypomagnesemia, mucopolysaccharidosis, achondroplasia, agenesis of corpus callosum, sotos syndrome and glutamic aciduria (4, 7, 27, 28).

## Differential Diagnosis

**1. Brain Atrophy:** Is the first and important differential diagnosis because of presence of subdural fluid collection in both conditions (3, 6, 7). In brain atrophy, CSF collection remains equal anteriorly and posteriorly but in BESS larger anterior convexity collections were seen (3, 5, 6). There is global widening of cerebral sulci in brain atrophy not associated with an increasing head circumference (28).

**2**. **Benign Familial megalencephaly:** This familial condition is benign and head circumference may be normal at birth but increases during infancy and ultimately should be above 98^th^ percentile. Development and neurologic examination are normal (28, 29). 

**3. Subdural fluid collection (SDE):** In this disorder there is CSF collection without hemorrhage in subdural space. SDE usually occurs in infants and young children after intracranial infections and less commonly after minor head injuries or neurosurgical operations (30, 31).

**4.** Other causes of communicating hydrocephaly such as; achondroplasia, choroid plexus papilloma, post meningitis hydrocephalus, basilar impression, Sotos syndrome and Glutaric aciduria type 1 (32). 

## Neuroimaging & Diagnostic work up

Neuroimaging findings are one of the criteria for the diagnosis of external hydrocephalus (7). The first step in confronting with an infant with macrocephaly is doing brain sonography via anterior fontanel (1, 33). This modality is fast, safe and non expensive tool used worldwide. Technical improvement in brain sonography have allowed more accurate visualization of the intracranial structures and may accurately evaluate ventricular size, extracerebral fluid collection and a significant number of a structural abnormality (33). Increased subarachnoid space was used as a diagnostic criterion. Three measurement tools for evaluation are sinocortical width (SCW), craniocortical width (CCW) and interhemispheric distance (IHD) (7, 5, 17). Ventricles are normal size or mildly enlarged without periventricular lucency. Normal ranges for CCW, SCW and IHD are from 4 mm to 10 mm, 2 mm to 10 mm and 6 mm to 8.5 mm, respectively (7, 9, 26, 34).

**Figure 2:**
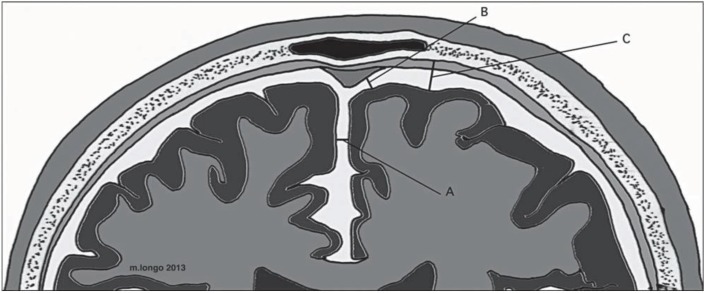
Main neuroradiological criteria for BESS evaluation: A) inter-hemispheric fissure; B) sino-cortical width; C) cranio-cortical width (Schematic view).

**Figure 3:**
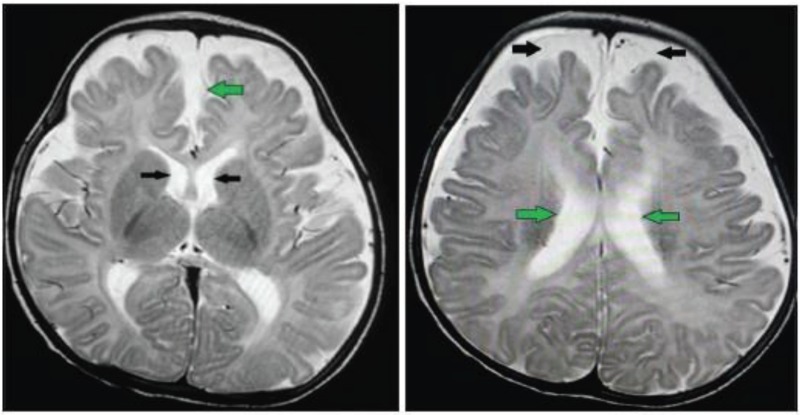
**BESS:** *(Left ):* Axial T2W MR image of the brain reveals mild prominence of both the lateral ventricles (black arrows) with increased anterior inter-hemispheric distance (green arrow).*(Right):* Axial T2W MR image of the same infant shows enlarged subarachnoid space along the anterior aspect of the brain (black arrows) with prominent of bilateral lateral ventricles (green arrows). Moreover, the anterior cranio-cortical distance (black arrows) is obviously increased.

The second step in evaluation of infant with abnormal brain sonography is brain CT scan or brain MRI (1, 7, 15, 17). Recently use of CT scan is limited because of its radiation and probable risk of malignancies especially in infants and young children (35, 36). MRI appears essential in the differential diagnosis between benign enlargement of subarachnoid space and subdural collection in infants and preferred to CT (37-39). CT and MRI without contrast are also important for evaluating the most common complications associated with external hydrocephalus (7, 38, 39) (Figure 3).

There is a very important note in diagnostic work up of BESS. Repeated imaging is unnecessary unless head growth deviates from the normal curve, neurological examination is abnormal, or social and language development are slow (2, 3, 13, 15, 17, 20, 33). 

**Final diagnostic note:** Patients with typical findings in brain ultrasonography suggestive of external hydrocephalus with normal neurodevelopment without any complications and focal neurologic findings *do not require* subsequent brain CT / MRI (40-42).

## Complications

The most common complication of BESS in infants and young children is increased risk of subdural hematoma after minimal or even without head trauma (3, 4, 12).

## Outcome

The head circumference usually stabilizes before the age of 18 months (2, 9). Measurements afterwards typically lie above but parallel to the upper (95^th^-98^th^) percentiles (2, 19). Overall, 11%-87% of these infants ending up with macrocephaly (10, 11). Mild gross motor delay with minimal language delay that decreased and disappeared within 1-4 years (15, 23). Most studies report in general normal physical and neurological findings on last follow-up (10, 20, 24, 44). Some studies report failure to reach developmental milestones especially in gross motor function (10, 11, 44). Mental retardation seems relatively rare (22, 23). The symptoms related to increased intracranial pressure which often can be seen initially, all appear to be absent at long-term follow-up (4). Generally the developmental delays are transient and children catch up milestones by the age of 2 yr (44, 45).

## Treatment

BESS is a self-limiting condition (3-5, 44, 45). A few old articles suggested use of a carbonic anhydrase inhibitor (Acetazolamide) for few weeks. Acetazolamide therapy for 4-8 wk in 125 mg/bd was recommended (22, 23, 45). This drug decreases CSF production (6, 23, 24, 45). There is no clear evidence of effectiveness of this agent in final outcome because of excellent nature of disease (25). Most patients do not need neurosurgical intervention (3, 4) and ventricular shunts (2, 18, 25, 45).

**In conclusion, **BESS is a benign self-limited condition, mostly were seen in infants. It is characterized by macrocephaly and enlargement of subarachnoid space with normal or mildly dilated ventricles. Neuroimaging study is necessary for establishing of diagnosis. First step for evaluation of infant with macrocephaly is brain sonography. If sonographic findings are matching with clinical findings, it is enough for diagnosis and further neuroimaging modalities are unnecessary. Second step for evaluation especially if any complication or suspicious underlying structural abnormality occurs is brain MRI.
